# Predicting and comparing COVID-19 risk perceptions across the Netherlands and Belgium: A cross-sectional survey among university students

**DOI:** 10.1371/journal.pone.0277417

**Published:** 2023-02-02

**Authors:** Ruben D. Vromans, Annemiek J. Linn, Nirvi Maru, Sara Pabian, Emiel J. Krahmer, Jeanine P. D. Guidry, Paul B. Perrin, Nadine Bol

**Affiliations:** 1 Department of Communication and Cognition, Tilburg Center for Cognition and Communication (TiCC), Tilburg School of Humanities and Digital Sciences, Tilburg University, Tilburg, The Netherlands; 2 Amsterdam School of Communication Research (ASCoR), University of Amsterdam, Amsterdam, The Netherlands; 3 Department of Communication Studies, Faculty of Social Sciences, University of Antwerp, Antwerp, Belgium; 4 Robertson School of Media and Culture, Virginia Commonwealth University, Richmond, VA, United States of America; 5 Department of Psychology, Virginia Commonwealth University, Richmond, VA, United States of America; University of Foggia: Universita degli Studi di Foggia, ITALY

## Abstract

People’s risk perception of COVID-19 is an important predictor for adopting protective behavior. Although risk perceptions, and factors influencing these, may vary between countries, less attention has been paid to differences between adjacent regions from neighboring countries. In the midst of the first wave of the corona outbreak (March-April-May 2020), we measured risk perceptions as perceived threat (consisting of perceived severity and susceptibility) among university students (*N* = 668) in two connected countries: the Netherlands and Belgium. Theory-based predictor variables included experiential, efficacy-related, socio-cultural, cognitive, and demographic factors. While demographic variables and country were not significant predictors of perceived threat level, all other constructs were. Personal and indirect experiences with COVID-19, as well as higher scores on personal (self) efficacy to carry out recommended preventive behaviors were all associated with higher perceived threat. However, low collective efficacy and lower levels of trust in government were both also significantly associated with higher perceived threat, as was a low level of “lack of COVID-19 knowledge”. These results hold implications for suitable risk communication strategies for increasing students’ COVID-19 risk perceptions.

## Introduction

Coronavirus disease (i.e., COVID-19) is an infectious disease caused by a newly discovered coronavirus [1], which emerged in Wuhan, China in late 2019 [2]. At the beginning of 2020, the virus was spreading rapidly throughout Europa, and also greatly affected The Netherlands and Belgium, two small neighboring countries in Western Europe, where the number of positive cases peaked for the first time in early April 2020 [3]. Hence, as elsewhere, the respective governments implemented drastic preventive measures to prevent the virus from spreading. These measures ranged from social distancing and hygiene advice to an (“intelligent”) lockdown, aimed at minimizing the spread of the virus and number of deaths while keeping the economy running as much as possible [4].

Many factors are expected to play a role in people’s willingness to adopt these preventive measures, including their risk perception of COVID-19 [5, 6]. Risk perception or perceived threat (a combination of perceived severity and perceived susceptibility) is a key determinant in nearly all traditional health behavior theories, including the Extended Parallel Processing Model [7], Health Belief Model [8, 9], or Protection Motivation Theory [10]. According to these theories, increases in perceived risks appear to be an important predictor of self-protecting behavior [11]. This relationship between risk perception and self-protection behavior has also been found during the first wave of the COVID-19 outbreak, including studies showing that people with greater levels of perceived severity of COVID-19 engaged more in self-protecting behaviors [12, 13]. Similarly, those who perceived themselves to be at risk for COVID-19 were more likely to adhere to preventive measures [14–16], or to take a vaccine that protects against COVID-19 [17].

However, risk perceptions are subjective by nature, and can be influenced by various other factors [18, 19]. According to Van der Linden’s risk perception model [20], peoples’ risk perceptions may be determined by experiential (i.e., affect, personal experience with a threat or hazard), socio-cultural (i.e., caring about others), cognitive (i.e., knowledge about a threat or hazard), and/or sociodemographic factors (i.e., gender, political orientation). Following this framework, an international survey study in ten countries across Europe, America, and Asia found that risk perception of COVID-19 was determined by factors such as direct personal experience with the virus, and peoples’ personal and collective efficacy [14]. Moreover, male participants generally showed lower risk perceptions, and people who had a higher trust in the government reported lower risk perceptions, while trust in science and medical practitioners were positively associated with higher risk perceptions. COVID-19 risk perceptions also differed between countries, with the highest levels of risk perceived in the United Kingdom compared to other countries [14].

While the Netherlands and Belgium experienced the start of the COVID-19 pandemic at the same time, and while at first glance the countries’ official response and actions appeared similar–schools, for example, were closed only a few days apart in mid-March 2020 –several interesting differences in approach and background were present [21]. Belgium instituted a hard lockdown, with strict rules on when people could leave their residence, while the Netherlands asked their residents to stay home as much as possible, and recommended limiting non-essential travel, but without as dramatically enforcing it [21, 22]. A background for this is that the Netherlands consistently ranks among the highest in national government trust (59% of respondents in a recent survey) while the Belgian population scores significantly lower (35% in a similar survey) [21]. These dynamics could have affected these two neighboring populations’ perception of the threat of the COVID-19 outbreak.

While university students initially were not seen as one of the highest risk groups for developing COVID-19, students are less likely to adhere to social distancing recommendations [23] and disease spread has been linked to shared events, parties, and shared alcohol consumption [24, 25]. University students also posed a risk to their families at home, who may have been more susceptible to the most severe consequences of COVID-19 [26].

1. Therefore, understanding factors that predict risk perception of COVID-19 among university students is an issue of importance, especially since it may yield practical guidance for risk communicators and policy makers during both this and future pandemic health crises. Using a theory-based approach [18, 20], the first aim of this paper is to explore which experiential, efficacy-related, socio-cultural, cognitive, and demographic factors predict peoples’ risk perception of COVID-19 in two neighboring countries, which leads to our first research question:
  What were the predictors of peoples’ risk perceptions of COVID-19 in the Netherlands and Belgium?
  The second exploratory aim of this paper is to explore regional differences on COVID-19 risk perceptions at the very beginning of the pandemic in two neighboring countries, the Netherlands and Belgium. For this second aim, we will address the following research question:
2. To what extent did peoples’ risk perceptions of COVID-19 differ between the Netherlands and Belgium?

## Materials and methods

### Study design and sample

For the purpose of this study, we collected a large convenience sample of university students. Surveys were distributed by faculty as well as student leaders. Most students are typically young, healthy, and have mild symptoms after being infected with COVID-19 [1], which can have a significant impact on perceived seriousness of the virus and their perceived likelihood of contracting the virus, and consequently the spread of COVID-19. Respondents were recruited through different recruitment messages that contained a Qualtrics link to the survey. Data were collected in the early phases of the first COVID-19 outbreak from March 30, 2020, until May 10, 2020 (due to the implementation of new governmental measures). We distributed the survey at three universities: two in the Netherlands (the University of Amsterdam and Tilburg University) and one in Belgium (University of Antwerp). Ethical approval was granted by the Amsterdam School of Communication Research (2020-PC-12104), the Research Ethics and Data Management Committee of the Tilburg School of Humanities and Digital Sciences of Tilburg University (ID REDC-2019-104b), and the Ethics Committee for the Social Sciences and Humanities (ID SHW-20-33).

A total of 1,100 responses were recorded by Qualtrics. Students were not required to participate or complete the survey and no incentive was provided. Consent was obtained within the Qualtrics structure, but before starting the survey itself. Responses were excluded if respondents did not complete the survey or if they completed the survey after May 10^th^. After applying these exclusion criteria, a total of 668 respondents (60.7%) were included in the sample for data analysis (*N*_NL_ = 383 (57%) and *N*_BE_ = 285 (43%)).

### Measures

#### Perceived threat

Perceived threat of COVID-19 consisted of two different, yet related aspects: perceived severity and perceived susceptibility [13, 19]. These were then combined into the average of both.

*Perceived severity* referred to people’s beliefs about the seriousness of COVID-19 [6], and was measured with eight items on a 5 point Likert scale (1 = ‘strongly disagree’, 5 = ‘strongly agree’). The items were a combination of items developed by Myers and Goodwin [27], modified for the current study, as well as items generated by the researchers. An example of an item includes “The novel coronavirus is a disease one can die from”. Cronbach’s alpha for the items was .74.

*Perceived susceptibility* referred to people’s beliefs about their chances of experiencing COVID-19 (6), and was assessed with three items on a 5 point Likert scale (1 = ‘strongly disagree’, 5 = ‘strongly agree’). These items were also taken from Myers and Goodwin [27] as well as researcher-generated items. An example item includes “It is likely that I will get the novel coronavirus”. Cronbach’s alpha for the items was .69.

#### Predictor variables

Similar to related studies on COVID-19 risk perceptions [14, 18, 20], we adopted a theory-based approach by including several predictor variables that ranged from experiential (personal and indirect experience), efficacy-related (personal and collective efficacy), socio-cultural (trust), cognitive (knowledge), and demographic (gender and age) factors.

We measured experience with COVID-19 by asking the respondent about their personal and indirect experience with the coronavirus. Respondents could either indicate that they had direct personal experience (i.e., the respondent suspected to be infected, was tested and diagnosed or was tested and was waiting for the results) or no personal experience with COVID-19 (i.e., the participant was not infected nor tested). Indirect experience was measured by asking respondents if someone in their immediate surrounding had been infected. Both variables were coded as 0 = ‘no experience’ and 1 = ‘experience’.

Personal efficacy (confidence in being able to adhere to required or recommended COVID-19 guidelines) was measured with one item, asking respondents how confident they were in adopting the preventive measures (e.g., social distancing, washing hands). Responses were measured on a 5-point Likert scale (1 = ‘little confident’, 5 = ‘very confident’). Collective efficacy (confidence that action as a population was possible and could affect change) was measured with one item in which respondents had to indicate the extent they believed that the Dutch or Belgian people could take any action to prevent getting infected (1 = ‘certainly not’, 5 = ‘certainly yes’).

For the socio-cultural factor, we measured respondents’ trust in the government, healthcare, and the situation being handled. Trust was measured with 5 items on a 5-point Likert scale (1 = ‘strongly disagree’, 5 = ‘strongly agree’). Since Cronbach’s alpha was adequate, but not optimal (.64), we also conducted the analyses with the 5 trust items separately. These secondary analyses revealed that the items “I am concerned about the impact of the coronavirus on my personal finances” and “I am confident that the government can address a coronavirus outbreak in my region” were indeed significantly and negatively correlated with perceived threat (respectively, β = -.096, *p* = .013 and β = -0.096, *p* = .029), indicating that the more students trust institutions such as the government, the lower their perceived threat. However, the items “I will receive appropriate healthcare if I become infected with the coronavirus” “Healthcare in my area is able to deal with the coronavirus” and “I can stay at home when I am sick (I can skip school or work)” were not significantly related to perceived threat, indicating that other aspects of trust, such as trust in the healthcare system, do not influence students’ perceived threat (respectively, β = -.025, *p* = .606, β = -.063, *p* = .224, and β = -.009, *p* = .825).

To assess respondents’ lack of knowledge about the coronavirus, we asked respondents to judge 28 statements about the coronavirus (1 = ‘certainly not true’, 5 = ‘certainly true’). Twenty-five of those statements were false, and three statements were true. An example of a false statement is “UV-light can kill the coronavirus”, and an example of a true statement is “Some symptoms of the coronavirus are comparable with those of the flu”. The mean lack of knowledge score was determined by computing the average score of the 28 items (with the three true-statements reverse-coded), with higher scores indicating lower levels of COVID-19 knowledge.

Gender was also included into the model, which was coded as 0 = ‘male’, and 1 = ‘female’, as was age in years.

### Statistical analysis

Descriptive analyses were conducted in order to describe the sample overall and also separately by country. Theoretical predictor variables were compared across the two countries in an exploratory manner using t-tests for continuous variables and chi-square tests for categorical variables. Dependent variables were distributed normally, confirmed by visual inspection of a Q-Q plot. We ran a hierarchical multiple linear regression wherein predicting perceived threat. In Step 1, the predictors were age and gender (which had been shown to differ between the two countries); Step 2 predictors included country and daily number of COVID-19 cases at the time; Step 3 predictors included the variables outlined above theorized to predict risk perceived threat. All statistical analyses were performed using SPSS version 28.0 (IBM Corporation, Somers, NY, USA). Tests were two-sided and considered statistically significant at p < .05.

## Results

The majority of respondents were female (78.9%), and, on average, 22.6 years old (*SD* = 5.11). When comparing the different regions, they significantly differed in age; the Dutch students were lightly but significantly younger (*M*_NL_ = 22.21, *SD* = 3.76) compared to the Belgian students (*M*_BE_ = 23.42, *SD* = 6.41), *t*(666) = –3.513, *p* < .001. In addition, Chi Square tests showed that the Dutch students were significantly more likely to be female compared to the Belgian students (χ^2^(1, 668) = 14.47, *p* < .001). Finally, collective efficacy (*t*(666) = –2.773, *p* = .006) and trust (*t*(666) = –3.935, *p* < .001) differed between the two countries (see Table 1).

**Table 1:** Descriptives of the sample (N = 668).

Characteristics	Entire sample (100.0%)		Netherlands (57.3%)		Belgium (42.7%)		
	*%*	*n*	*%*	*n*	*%*	*n*	*p*-value
Gender							< .001*
Male	21.1	141	15.9	61	28.1	80	
Female	78.9	527	84.1	322	71.9	205	
Experience							
Personal experience	15.3	102	17.0	65	13.0	37	.156
Indirect experience	49.9	333	52.2	200	46.7	133	.156
Continuous predictors	*M*	*SD*	*M*	*SD*	*M*	*SD*	
Age (years)	22.6	5.1	22.0	3.8	23.4	6.4	< .001*
Personal (self) efficacy	4.21	.49	4.21	.5	4.21	.47	.904
Collective efficacy	3.76	1.01	3.66	1.01	3.88	1.01	.006*
Trust	3.71	.58	3.64	.56	3.81	.6	< .001*
Lack of knowledge	2.15	.31	2.15	.33	2.14	.29	.454

### Predictors of risk perceptions of COVID-19 (RQ1)

In the linear regression investigating predictors of perceived threat of COVID-19 among university students in the Netherlands and Belgium, Step 1 was not statistically significant, *F*(2, 665) = 1.058, *p* = .348, and only explained .3% of the variance in perceived threat. Within the step, neither predictor was significant (Table 2). Step 2 also was not statistically significant, *F*(4, 663) = 2.197, *p* = .068, and explained 1.3% of the variance in perceived threat. Within this step, only country was a significant predictor such that participants from the Netherlands reported higher threat perception than participants from Belgium (Table 2). With the addition of the other theoretical variables in Step 3, the overall model was significant, *F*(10, 657) = 6.941, *p* < .001, and explained 9.6% of the variance in perceived threat. Within the model, the unique predictors of increased threat were personal experience, indirect experience, personal efficacy, collective efficacy, trust, and lack of knowledge of COVID-19. The presence of personal and indirect experiences with COVID-19 was associated with higher perceived threat, and higher scores on personal (self) efficacy to carry out recommended preventive behaviors was also associated with higher perceived threat. However, lower scores on collective efficacy and trust in government were both associated with higher perceived threat, and lower scores on lack of knowledge (in other words, increase correct knowledge) was also associated with higher perceived threat.

**Table 2:** Hierarchical multiple regression predicting perceived threat.

Variable	Step 1 Beta	*p*-value	Step 2 Beta	*p*-value	Step 3 Beta	*p*-value
Step 1						
Age	.038	.325	.049	.211	.026	.501
Gender: Female (Ref: Male)	.045	.245	.034	.393	.011	.774
Step 2						
Country: Netherlands (Ref: Belgium)			.087	.026*	.039	.313
Daily COVID-19 cases			-.086	.193	-.104	.105
Step 3						
Personal experience					.121	.001*
Indirect experience					.088	.020*
Personal (self) efficacy					.091	.013*
Collective efficacy					-.105	.008*
Trust					-.186	< .001*
Lack of knowledge					-.081	.024*

* *p* < .05.

### Country differences related to COVID-19 perceived threat (RQ2)

In the final hierarchical regression model, country was no longer a significant predictor of perceived threat and was likely explained by the inclusion of the theoretical predictors that differed between countries (e.g., collective efficacy and trust).

## Discussion

In the midst of the first wave of the corona outbreak, we cross-sectionally surveyed and compared university students’ risk perceptions of COVID-19 across two closely related European countries: the Netherlands and Belgium. While other studies have frequently found differences in basic demographic variables related to perceived COVID-19 risk or threat, such as women reporting a higher perceived risk [14], these differences did not materialize in the current study, even though Belgian students in this sample were slightly older, and Dutch students reported a higher percentage of females. A possible reason is that university are considered to be a more homogenous population with a closer, younger age range and a similar stage in life [28].

Furthermore, while we may have expected some differences between the two countries based on the difference in government approach in the early months of the pandemic [4, 21, 22], country only mattered to a small degree in predicting perceived COVID-19 threat, with small differences between the Netherlands and Belgium in the area of trust and collective efficacy. However, this minor effect disappeared once those factors and the other theoretical constructs were added to the regression model. While there were distinct differences between the two countries, they were perhaps more alike after all, in the early months of the pandemic, at least at the university student population level.

Perhaps most importantly, the theoretical constructs of risk perception used in this study [18, 20]–personal experience, indirect experience, personal (self) efficacy, collective efficacy, trust in government, and lack of knowledge–were significant predictors of perceived threat of COVID-19. More specifically, both personal and indirect experiences with COVID-19 were predictive of higher perceived threat. This could point to the importance of using narrative experiences of student peers to communicate the threat of COVID-19, something public health communication specialists should consider.

In addition, low collective efficacy scores–the confidence that society as a collective could take action to successfully address the COVID-19 pandemic–as well as low levels or trust in the government’s ability to play a role in this were both associated with higher perceived threat. This may be explained, at least in part, by the swift changes in the pandemic landscape during the administration of this survey: both the number of cases and number of deaths increased quickly in both countries, even while fairly restrictive prevention measures were either mandated or strongly recommended, potentially eroding trust in government and society to stem the tide of this outbreak. This points to the importance of balanced communication in a crisis such as COVID-19 [29]. The lessons learned in both these early pandemic days as well as the remainder of the pandemic should be distilled and processed for all target audiences, but perhaps especially for one that at the start of the pandemic was seen as one of relatively low risk.

Perhaps the most encouraging and at the same time concerning finding was that lower scores in lack of knowledge were associated with higher perceived COVID-19 threat–in other words, the more accurate a student’s knowledge base about COVID-19 was, the more dangerous they perceived COVID-19 to be. This of course makes a powerful case for public health education in the midst of a pandemic such as this one. However, the inverse is concerning: Many of the “lack of knowledge” statements are either pieces of misinformation meant to provide a false sense of security (such as the ingesting of garlic protecting against the virus) or plainly dangerous (such as the statement that gargling with or swallowing bleach will get rid of the virus, or perhaps a little less extreme: Antibiotics both prevent and cure COVID-19). A lack of knowledge can therefore be dangerous in and of itself, but the results show it is also associated with a lower perceived threat. Considering the effect of misinformation, later in the pandemic, on COVID-19 vaccination uptake and beliefs in treatments that remain unproven [30], an emphasis should continue to be on education about this virus and now its variants.

Our study is not without limitations. Although we distributed our survey in two adjacent European countries among the same homogenous sample of university students, we cannot be sure whether those students were living in that specific country continuously. Another limitation was, of course, that the cross-sectional convenience sample precludes us from generalizing the findings to the larger population of students. Moreover, we could consider the use of single item measures for some of the theoretical constructs (e.g., personal efficacy) a limitation, since construct validity could not be appropriately established. Finally, even though we found that lower levels of trust (in government) predicted higher perceived threat, we acknowledge and realize that not all facets of trust (e.g., in healthcare) showed this relation with perceived threat in our study.

In conclusion, and in line with previous research, our results show that students’ perceived threat of COVID-19 was consistently predicted by theoretical constructs including experiential, efficacy, and socio-cultural (trust) factors across regions [14, 16]. This provides an encouraging indication that these constructs can and should be used in public health education campaigns. However, we also found that previously established demographic factors such as gender and age did not predict students’ level of perceived threat. Further research is needed on suitable risk communication strategies for students and young adults to increase their COVID-19 risk perceptions and to be prepared for future variants as well as other outbreaks.
